# The change of teeth color, whiteness variations and its psychosocial and self-perception effects when using low vs. high concentration bleaching gels: a one-year follow-up

**DOI:** 10.1186/s12903-020-01244-x

**Published:** 2020-09-11

**Authors:** J. Estay, P. Angel, C. Bersezio, M. Tonetto, G. Jorquera, M. Peña, E. Fernández

**Affiliations:** 1 Department of Restorative Dentistry - Faculty of Dentistry, University of Chile, Sergio Livingstone Pohlhammer 943, Independencia, Santiago Chile; 2 Postgraduate Program in Integrated Dental Sciences, University of Cuiabá, Cuiabá, Brazil; 3 Department of Prosthodontics, College of Dentistry, Universidad de Los Andes, Santiago, Chile; 4 Instituto de Ciencias Biomédicas - Universidad Autónoma de Chile, Av. Pedro de Valdivia 425, Providencia, Santiago Chile

**Keywords:** Bleaching, Randomized clinical trial, Low concentration, OHIP-14, PIDAQ

## Abstract

**Background:**

Dental bleaching in traditional concentrations generates greater sensitivity. In this respect, new systems of lower concentration of hydrogen peroxide for tooth bleaching appeared, with color stability unknown over time. The aim of this study was to compare the change and stability of color with low-concentration (6%) hydrogen peroxide gel in an in-office bleaching setting relative to conventional 37.5% gel, including their effects on psychosocial and esthetic self-perception, after 1 year.

**Methods:**

Patients (*n* = 25) were assessed at 12 months post bleaching treatment (whitening with 6% chemo-activated alkaline formula gel versus 37.5% traditional concentration gel). Color changes were measured objectively using total variation in color (ΔE), and subjectively using Vita Classical and Vita Bleached scale (ΔSGU) by calibrated evaluators (Kappa = 0.85). The Psychosocial Impact of Dental Aesthetics Questionnaire (PIDAQ) and Oral Health Impact Profile (OHIP-14) aesthetic questionnaires were used to measure the self-perception and the psychosocial impact of the bleaching protocols.

**Results:**

The effect (ΔE) of 37.5% HP (8.37 ± 2.73) was significantly better than that of 6% HP (5.27 ± 2.53) in terms of color rebound after 1 year of follow-up. There were significant differences in psychosocial impact and esthetic self-perception measurements prior to bleaching versus one-year post-whitening time points; positive effects were maintained.

**Conclusions:**

Low concentration (6%) achieved effective bleaching with good stability after 1 year, accompanied by a positive psychosocial impact and enhanced self-perception at follow-up.

**Trial registration:**

NCT03217994 (before enrollment of the first participant). Data register: July 14, 2017.

## Background

Teeth whitening is a safe and widely used procedure which is frequently requested by patients searching for aesthetic improvement, despite some reported biological side effects [1]. As a routine procedure, the quantification of tooth whitening and the efficacy of its effects has turned into a crucial concern.

Traditionally, dentists determine the color of human teeth via visual comparison to a reference standard set called a shade guide. Alternatively, the instrumental assessment provides quantitative and objective data which through the use of some indexes widely used allow for proper interpretation of them. The list of indexes includes the CIE whiteness index WIC, the whiteness index according to ASTM E-313-73 WI, and the Z% index. A whiteness formula (WIO) that optimizes the original CIE whiteness formula (WIC) has been developed, rendering the best performance for the prediction of tooth whiteness [2, 3]. Gerlach considered an index that tried to explain better the differences in whiteness based on the difference in maximum luminosity (L-100) and incorporating it into a formula similar to that of delta E to explain its delta W [4]. Pérez et al. [5] proposed a new index (WID) that outperformed previous indices when it comes to evaluation of whiteness in dentistry by providing a better correlation with visual perception.

Regarding the bleaching product, traditionally a chemically activated gel of low concentration of hydrogen peroxide has shown lower efficiency than a higher concentration one; in the same application time, however, a new alkaline formulation has shown color changes of over 5 ∆E units [6]. Home whitening which is considered the conventional and gold standard method uses 10% or 16% carbamide peroxide, which represents low concentrations of hydrogen peroxide but is performed for a much longer time than in-office whitening.

The low-concentration product in office whitening has demonstrated promising results including high efficacy with very low postoperative sensitivity. The alkalinity of this product would play a key role accelerating the chemical reaction and improving the effectiveness when compared to others systems of similar concentration [7, 8].

At the same time, despite their importance, reports about longevity [9–11], and the relationship between dental whitening and quality of life [12–14] are limited, besides relatively few clinical studies have assessed the effectiveness, and psychological effects of tooth whitening in a long-term follow-up [15, 16]. In this sense, it is essential to study patients’ self-perception concerning to whitening psychosocial impact and whitening duration [17]. Recent studies suggest that extra and intracoronal tooth whitening can produce positive psychosocial outcomes and increase the self-image of the patients [18]. However, there are few reports [15, 19] of the psychosocial and self-perception effects of low concentration gels (6% hydrogen peroxide) in a prospective and longitudinal follow-up study.

This study compared the stability and color rebound at 12 months after using a low concentration alkaline 6% hydrogen peroxide gel versus a conventional 37.5% gel in an office whitening procedure. The color was assessed using regression by standard methodologies and whiteness indexes (WI and WI_D_). Additionally, the psychosocial effects and effects on self-perception were evaluated during 1 year of follow up.

This study tested three null hypotheses: 1) there will be no color rebound after 1 year of follow-up in patients treated either with 6% or 35% hydrogen peroxide gel and 2) The color difference of the 2 whitening systems will be maintained at 1 year of follow-up and 3) there will be no variation in the psychosocial and self-perception effects in the patients, after teeth whitening, a year later.

## Methods

### Sample and ethical approval

**Fig. 1:**
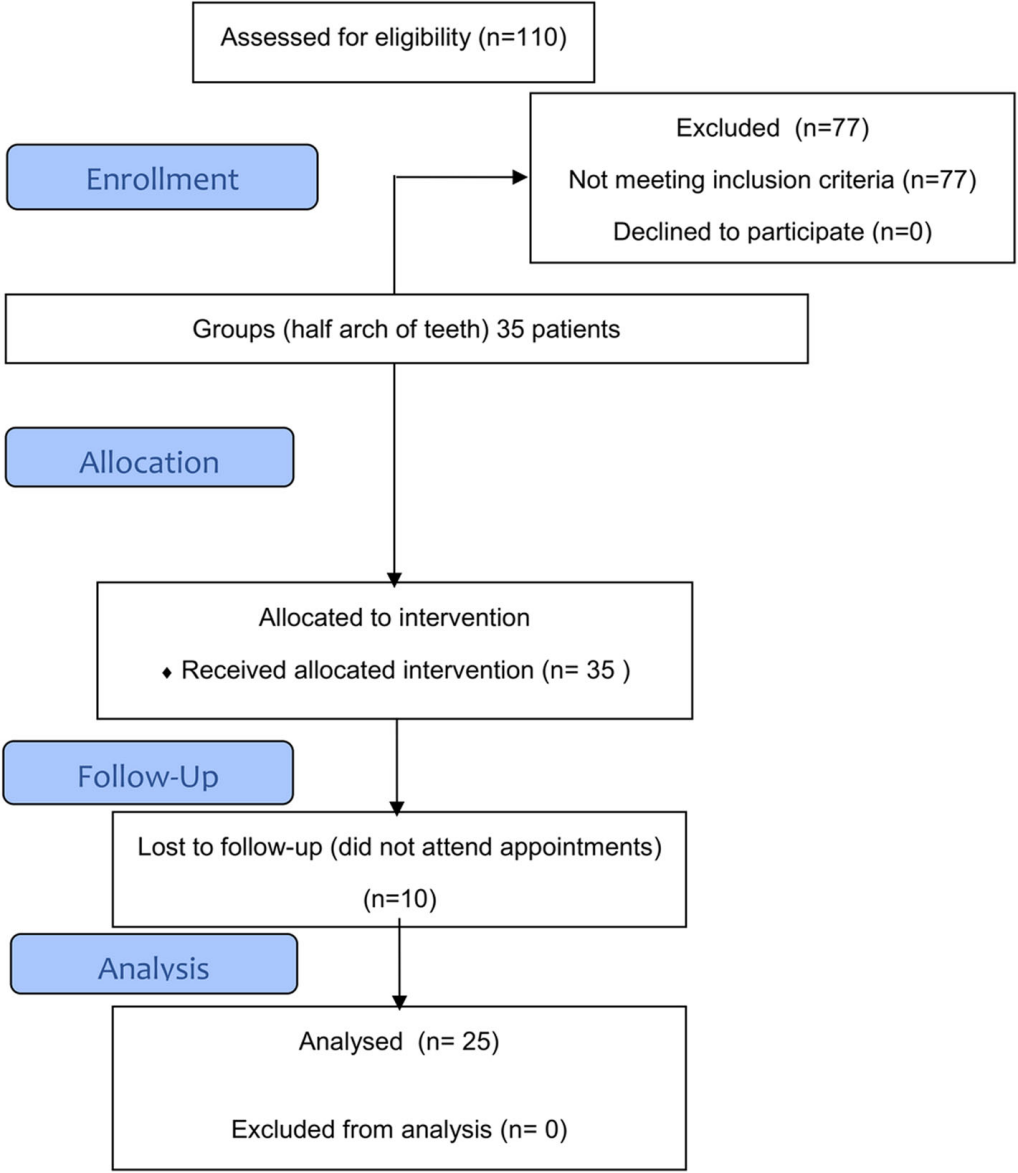
Flow diagram of the clinical trial

This study was a randomized and prospective double-blind clinical trial with 1 year of follow-up. The design is shown in Fig. 1 and follows the CONSORT (Consolidated Standards of Reporting Trials) recommendations and the principles of the Helsinki Convention. The study was approved by the local Committee of Ethics approval number (15/001) and registered in ClinicalTrials.gov (NCT03217994) on July 14, 2017. All Participants signed an informed consent form approved by the Ethics Committee of the Faculty of Dentistry of the University of Chile.

### Selection of the sample

Thirty-five patients were recruited from the Faculty of Dentistry of the University of Chile. These patients had asked for a whitening treatment and volunteered to participate in the study by signing an informed consent form approved by the Ethics Committee of the Faculty of Dentistry.

All of these patients met the following inclusion criteria:

- Age 18 or older (both sexes).
- At least six upper healthy frontal teeth, with no restorations or crowns.
- A color A3 or darker (using the Vita classical scale) as determined using a spectrophotometer (Vita Easy Shade® Compact, Vita Zahnfabrik) in the middle third on the buccal surface of central incisors.
- The following exclusion criteria were used:
- Pregnancy or lactation.
- Bruxism or tooth sensitivity.
- Teeth with prior whitening treatment (either at home or in-office).
- Teeth with visible dental cracks, developmental defects, or tetracycline or fluorosis discoloration.
- Presence of non-carious cervical lesions
- Nonvital discolored teeth.

### Study design

Patients who were evaluated and were excluded due to having any pathology that prevented them from entering the study (such as caries, periodontal disease, or tooth sensitivity) were referred to the dental clinic of the Faculty of Dentistry at the local University for proper treatment.

The sample size was determined following similar studies [20] with a significance level of 5% and a statistical power (1-β) of 0.90. As a result, it was estimated a minimum of 32 patients.

Since it was expected a 5% dropout; the initial number of participants was 35 individuals. Treatments were carried out in the clinic of the Faculty of Dentistry; the researchers monitored the participants.

The study used a split-mouth randomized design with a 37.5% hydrogen peroxide (Polaoffice + 37.5%, SDI, Victoria, Australia) (pH = 7.0) [7, 8] and a 6% hydrogen peroxide (Polaoffice + 6%, SDI, Victoria, Australia) (pH = 8.0) as whitening agents. Both products were used in each participant, applying the 6% to one hemi-arcade (canine, central, and lateral incisors) and the 37.5% to the other. The whitening systems were assigned using SPSS 21 software (SPSS, IBM, New York, USA). To blind the operators, each product was properly masked with coded labels. Auto-mix syringes of Polaoffice + in the office teeth whitening system were used (SDI Limited). The syringes contained hydrogen peroxide in concentration of 37.5% or 6% in the form of a thixotropic gel with similar features of color and viscosity.

Different operators than those who performed the whitening procedure conducted all color measurements in both upper central incisors. The follow-up was performed similarly.

### Preliminary phase

The color of each upper central incisor was assessed by comparison with shade tabs of two commercially available dental shade guides (Vita classical Guide and Vita Bleached guide, Vita Zahnfabrik) and objectively with spectrophotometer (Vita Easy Shade® Compact, Vita Zahnfabrik) that was previously calibrated according to the manufacturer instructions. To standardize this evaluation, a silicone matrix was made (Zetaplus, Zhermack, Rovigo, Italy) with a 6 mm-diameter window on the buccal surface that allowed the positioning of the tip of the spectrophotometer on the middle third of the labial surface of the teeth.

### Intervention: whitening protocol

Each participant had two whitening sessions separated by an interval of 1 week. At the beginning of each session, dental prophylaxis was performed using a dental brush and stone pumice with water at low speed. A plastic lip retractor and a gingival barrier (Gingival barrier; SDI Limited, Victoria, Australia) were used to protect the soft tissues. Whitening gels were applied evenly to each hemi-arcade on their vestibular surface.

The Protocol included three applications of the whitening gel for 12 min (36 min each session), to standardize the protocol between both gels. Between each application, the gel was removed with rolls of cotton moistened with water and then the teeth were dried carefully. At the end of the third application, the gel was removed using copious amounts of water, and the gingival barrier and lip retractor were removed. The patients received indications and were scheduled to the next visit.

### Controls

At the end of the first session, tooth color was measured subjectively (Color scales) and objectively with a spectrophotometer (Vita Easy Shade® Compact, Vita Zahnfabrik) [21]. The measurements were repeated after a week, a month, 6 months and a year after whitening.

### Color evaluation

#### Subjective extraction and treatment data

The color was assessed visually under standardized light conditions (same place, time, natural light source, all assessments were performed between 10:00 AM and 3:00 PM) by two previously calibrated operators, who showed a previous agreement (Visual Scales) of at least 85% as determined using weighted k-statistics. The viewing geometry, object-observer distance, visual angle, and background color were held constant. Each operator evaluated three times each tooth. If both operators coincided in their selection, the determined value remained as definitive, if there was a discrepancy, a calibrated third operator (professor of restorative dentistry) defined between both colors.

The Vita classical Guide (Vita Classic, Vita Zahnfabrik) and Bleached guide (Vita Bleached Guide, Vita Zahnfabrik) were used for subjective evaluation using the tab arrangement proposed by Ontiveros and Paravina [22]. The observers assessed the color of both central incisors at the start of the study, at each session of whitening, and 1 week, 1 month, 6 months and one-year post-treatment. The color was recorded on the middle third of the labial surface as established by the guidelines of the American Dental Association [23]. The difference in tooth color was calculated as the number of units that the tooth changed according to the shade guide arrangement (ΔSGU).

#### Objective treatment data

The color was measured objectively with the spectrophotometer, in the same way as described in the preliminary phase. Objective data were evaluated according to the three axes of the CIELAB system (L *, a * and b *). The ΔE was calculated as the Euclidean distance as follows:

ΔE = [(ΔL) ^2^ + (Δa) ^2^ + (Δb) ^2^] ^1/2^.

The variation on each parameter at different time points was calculated about the initial value (i.e., the measurement of color before the first session of whitening).

Also, the color difference was calculated by means of the CIEDE2000 formula proposed by Luo in 2001 [24], Whiteness Index proposed by Gerlach in 2002 [4] and WI_D_ by Pérez in 2016 [5].

### Self-perception and psychosocial impact assessment

Before whitening treatment, participants completed two questionnaires: the psychosocial impact dental aesthetic questionnaire (PIDAQ) and the oral health impact profile (OHIP-14). These questionnaires were repeated after a week, 1, 6 and 12 months post whitening treatment. They were completed under the supervision of an examiner who was available to answer every question.

The PIDAQ is psychometric testing to measure the psychosocial consequences of dental aesthetics [25]. It consists of 23 items on a five-point Likert-type scale from 0 (total disagreement) and to 4 (complete agreement). A patient may receive a total score of 0 to 72 points. The evaluation is also divided into four subscales: one positive (dental confidence [six questions]) and three negative (psychological impact [eight questions], aesthetic concern [three questions], and social impact [six questions]). A more positive subscale score indicates greater confidence in itself, while higher scores on the negative subscales indicate the adverse effects of cosmetic dentistry.

The OHIP-14 is an assessment used to evaluate the aesthetic perception [26]. The survey is scored on a five-point Likert-type scale. Each option partners with a score: very often (4), often (3), from time to time (2), almost never (1), or never or not (0). A higher score indicates poor patient self-perception of the cosmetic dentistry. To calculate the score of OHIP-14 for each patient, we added the total score of 14 questions to generate a total score between 0 and 56 points.

### Statistical analysis

The data were tabulated, and their normal distribution was analyzed using the Shapiro-Wilk test. The Mann-Whitney test was used to compare the efficiency of the results between groups, whereas, for comparisons between two assessment times and to assess color rebound in each group the Wilcoxon and T-Student tests were used.

PIDAQ and OHIP-14 test scores were determined, and the results for each time point were compared using the Wilcoxon test. Data were statistically analyzed using SPSS 21.0 (Lead Technologies INc., Charlotte, NC, USA). The data were considered statistically significant when *p* < 0.05.

## Results

### Features of the sample

After 1 year of follow-up, twenty-five participants were evaluated. The average age was 27.11 years old (total range 20/54 SD = 7.5) and 13 were men and 12 women; other features are summarized in Table 1.

**Table 1:** Participants’ characteristics at baseline

Baseline features	Groups	
	HP 37.5%	HP 6%
Age (years; mean ± SD)	27 ± 7.5	
Minimum age (years)	20	
Maximum age (years)	54	
Male (%)	52%	
*L (mean ± SD)	84.41 ± 2.95	86.17 ± 2.81
*a (mean ± SD)	0.08 ± 1.06	0.15 ± 1.24
*b (mean ± SD)	27.96 ± 3.18	28.42 ± 3.68
Baseline Vita bleach SGU median (min:max)	9 (7:11)	9 (7:11)
Baseline Vita classical SGU median (min:max)	9 (9:12)	9 (9:12)

*SD* Standard deviation

#### Effectiveness values

One month after treatment the 37.5% hydrogen peroxide group has an effectiveness of 3ΔSGU and the 6% hydrogen peroxide group at 2ΔSGU as measured with the VITA Bleached guide. In the objective measurement, the group of 37% hydrogen peroxide a month after treatment had a value of 9.05 ± 2.74ΔE and the 6% group had a value of 5.08 ± 2.11ΔE. At 1 year, the ΔE and ΔSGU changes (< 10%) values were similar, the color rebound was considered minimal and was not statistically significant (Tables 2, 3 and 4).

**Table 2:** Comparison of ΔSGU values by Vita Classic guide and Vita Bleach Guide 3D-Master at different times. The median, minimum and maximum values are shown

Assessment points	Color change by ΔSGU Vita Classical			Color change by ΔSGU Vita Bleachguide		
	37.5% Hydrogen Peroxide	6% Hydrogen Peroxide	Mann-Whitney test*	37.5% Hydrogen Peroxide	6% Hydrogen Peroxide	Mann-Whitney test*
Baseline vs. 1-wk after bleaching	7 (2:9)	6 (2:9)	0.205	3.5 (1:6)	3 (2:6)	0.156
Baseline vs. 1-mth after bleaching	7 (2:9)	6 (1:9)	0.054	3 (1:6)	2 (0:5)	0.040
Baseline vs. 6-mths after bleaching	7 (3:8)	6 (1:8)	0.055	3 (2:5)	2 (0:4)	0.004
Baseline vs. 12-mths after bleaching	6 (1:8)**	5 (0:7)**	0.033	3 (0:5)**	2 (0:4)**	0.002

* for comparison between both groups in each assessment time; ** for comparison between two assessment time (1-month vs 12-month after bleaching) in each group. No significant difference was found (Wilcoxon test; *p* > 0.05).** for comparison between two assessment time (1-month vs 12-month after bleaching) in each group. No significant difference was found (Student t test for paired sample; *p* > 0.05)

**Table 3:** Comparison of ΔE values and Color change using CIEDE2000 formula, with data from the Vita Easyshade spectrophotometer measurements at different times expressed as mean and SD

Assessment points	Color change by ΔE			Color change by ΔCIEDE2000		
	37.5% Hydrogen Peroxide	6% Hydrogen Peroxide	Mann-Whitney test*	37.5% Hydrogen Peroxide	6% Hydrogen Peroxide	Mann-Whitney test*
Baseline vs. 1-wk after bleaching	8.67 ± 2.61	5.59 ± 3.41	0.000	5.10 ± 1.57	3.22 ± 1.90	0.000
Baseline vs. 1-mth after bleaching	9.05 ± 2.74	5.08 ± 2.11	0.000	5.37 ± 1.61	2.94 ± 1.15	0.000
Baseline vs. 6-mths after bleaching	8.01 ± 2.84	5.07 ± 2.98	0.001	4.73 ± 1.59	2.87 ± 1.61	0.000
Baseline vs. 12-mths after bleaching	8.37 ± 2.73**	5.27 ± 2.53**	0.000	4.92 ± 1.55**	3.07 ± 1.36**	0.000

* for comparison between both groups in each assessment time; ** for comparison between two assessment time (1-month vs 12-month after bleaching) in each group. No significant difference was found (Wilcoxon test; *p* > 0.05).** for comparison between two assessment time (1-month vs 12-month after bleaching) in each group. No significant difference was found (Student t test for paired sample; *p* > 0.05)

**Table 4:** Color change using WI and WI_D_ values, with data from the Vita Easyshade spectrophotometer measurements, for each group of treatment in different time points. The mean, standard deviation, and statistical analysis are displayed

Assessment points	Whiteness Index values			WId values		
	37.5% Hydrogen Peroxide	6% Hydrogen Peroxide	Mann-Whitney test*	37.5% Hydrogen Peroxide	6% Hydrogen Peroxide	Mann-Whitney test*
Baseline	32.19 ± 2.88	31.79 ± 3.39	0.479	11.93 ± 6.04	12.23 ± 7.65	0.789
1-wk after bleaching	24.91 ± 2.85	27.63 ± 4.07	0.018	25.44 ± 4.74^a^	20.12 ± 7.83^a^	0.011
1-mth after bleaching	24.65 ± 2.94	28.20 ± 3.39	0.001	25.53 ± 6.62^a^	18.67 ± 6.87^a^	0.001
6-mths after bleaching	25.53 ± 2.63	29.31 ± 3.78	0.000	24.12 ± 4.80^a^	16.18 ± 6.86^ab^	0.000
12-mths after bleaching	24.89 ± 2.69**	27.81 ± 3.31**	0.003	24.26 ± 4.66^a^	18,96 ± 7.13^a^	0.003

* for comparison between both groups in each assessment time; ** for comparison between two assessment time (1-month vs 12-month after bleaching) in each group. No significant difference was found (Wilcoxon test; *p* > 0.05).** for comparison between two assessment time (1-month vs 12-month after bleaching) in each group. No significant difference was found (Student t test for paired sample; *p* > 0.05)

The results for the CIEDE2000 formula (Table 3) and Whiteness Index/ WI_D_ (Table 4) showed a significant difference for both groups (*p* > 0.05) in all timepoints after bleaching. The difference showed by WI_D_ is higher than WI, but the difference (p > 0.05) at different time points is maintained.

#### PIDAQ and OHIP-14

In PIDAQ all the factors were statistically significant at 1 year of follow-up (*p* < 0.05) (Table 5). In OHIP-14 the changes remained statistically significant at 1 year of follow-up (p < 0.05) for all the factors and the overall sum (Table 6).

**Table 5:** PIDAQ results at different time points. A: Statistically significant differences (Wilcoxon test, < 0.05) versus baseline. Expressed in median values (minimum/maximum). B: Statistically significant differences (Wilcoxon test, *p* < 0.05) versus 1 week after bleaching

	Time Points				
Dimension	Baseline	1 week	1 month	6 months	12 months
		after bleaching	after bleaching	after bleaching	after bleaching
Dental Self-Confidence	16	23	22	22	23
	(11:28)	(15:28) A	(16:29) A	(13:27) A	(14:29) A
Social Impact	17	16	12	13	10
	(9:34)	(8:27) A	(8:26) AB	(8:24) AB	(8:24) AB
Psychological Impact	19	15	13	14	11
	(8:26)	(6:22) A	(6:23) A	(6: 22) A	(6:19) AB
Esthetic Concern	7	6	5	3	3
	(3:15)	(3:10) A	(3:10) A	(3: 9) A	(3:10) A

**Table 6:** OHIP results at different time points

	Time Points				
Dimension	Baseline	1 week after bleaching	1 month	6 months	12 months
			after bleaching	after bleaching	after bleaching
Functional limitation	3 (0:7)	3 (0:6) A	2 (0:6) A	2 (0:5) AB	2 (0:6) A
Physical pain	3 (0:7)	2 (0:4) A	2 (0:6) A	2 (0:6) A	2 (0:5) A
Psychological discomfort	3 (0:7)	3 (0:5)	3 (0:5) AB	3 (0:6)	3 (1:5) A
Physical disability	1 (0:4)	0 (0:3) A	1 (0:2) A	4 (0:6) A	0 (0:3) A
Psychological disability	1 (0:5)	0 (0:3) A	0 (0:3) A	5 (0:6) A	0 (0:3) A
Social disability	0 (0:4)	0 (0:3) A	0 (0:2) A	6 (0:6) A	0 (0:2) A
Handicap	0 (0:4)	0 (0:3) A	0 (0:3) A	7 (0:6) A	0 (0:2) A
Sum	14 (6:31)	10 (3:19) A	10 (0:19) A	8 (0:6) A	8 (2:22) A

Expressed in median values (minimum/maximum). A: Statistically significant difference (Wilcoxon test, < 0.05) versus baseline. B: Statistically significant differences versus previous time-point (Wilcoxon test, *p* < 0.05)

## Discussion

This study evaluated color and whiteness variations as well as their rebound after using a low concentration (6%) of hydrogen peroxide gel compared to a standard 37.5% hydrogen peroxide gel in a split-mouth design. Both gels were effective and did not show a significant clinical rebound after 1 year of follow-up; however, each of them had different effectiveness. The positive psychosocial impact and aesthetic self-perception remained without changes up to 1 year. Thus, the first null hypothesis was accepted because the rebound was insignificant both in subjective and objective assessments. The second null hypothesis was accepted because the difference of color between both systems remained. Moreover, the third hypothesis was rejected because the positive psychosocial impact and aesthetic self-perceptions remained stable after 12 months of whitening.

Low concentrations of alkaline hydrogen peroxide gel showed good stability without significant rebounds in tooth color during the extension of this study, which could mean that during the first year, the concentration of hydrogen peroxide would not have a substantial impact on color rebound. Although the initial color differences between both hemi-arcades remained, the patients did not perceive them negatively, probably due to the perception threshold of each patient [27, 28]. That is, they were not able by some studies to discriminate between less than three ΔE [29]. The local Ethics Committee required that the researchers retreated any hemi-arcades to match the color if any patient noticed a difference; however, no patient raised this issue. This point denotes the critical difference between subjective and objective measurements, to the limit of having a cohort of patients, who did not perceive major differences between their bleached hemi-arcades, which showed statistical differences in objective measurements. This phenomenon should help to understand that from the patients’ perspective, an effective whitening can be achieved with a gel of low concentration, which also exhibited good stability at the one-year control, even when measured by CIEDE2000 and Whitening indexes (WI and WI_D_), which according to the literature, reflect more precisely the rebound of whitening treatments [3, 24].

The importance of aesthetic dentistry in recent years has led to technological developments that improve dental aspects [30]. The dental appearance exerts a powerful aesthetic influence on patients. Thus, it is essential to understand the psychosocial impact of treatment [31, 32]. However, these effects are poorly understood. The new definition of oral health recently declared by the FDI includes the psychological aspects of the patients. Therefore a procedure that improves these aspects is relevant for the mental health of them [33]. The results of this study showed that there were significant changes in PIDAQ and OHIP-14 scores after whitening, indicating that whitening had a positive impact on the subjects’ psychosocial and aesthetic perceptions. This effect remained for the duration of the study. The evaluation of the impact on the patient’s life is on the effect of whitening itself, with this experimental design, it cannot be evaluated whether the concentration difference has a different effect on the subject’s quality of life.

Dental self-confidence was measured using the PIDAQ. This positive subscale measures the influence of esthetic dentistry on an individual’s self-perceived image. The appearance of the mouth and smile play a vital role in the evaluation of the facial appeal. The results suggest that extra-coronal tooth whitening increases dental confidence. This finding shows that this factor is associated with more favorable attitudes toward oral health and a higher degree of satisfaction with regard self-perception [17, 25].

The PIDAQ also measures three negative psychosocial impact dimensions: social impact, psychological impact, and aesthetic concerns. Social impact evaluates potential problems that an individual has in social situations due to the unfavorable subjective aspects of their teeth. The psychological impact evaluates feelings of inferiority or unhappiness that an individual has when compared to others. Aesthetic concern includes data referring to the concern or disapproval of dental appearance an individual has when that person faces the mirror or view photographs or videos of themselves [32]. The results show a decrease in these scores at one-year post-whitening when compared to the baseline. Therefore, extra-coronal tooth whitening generates a positive psychosocial effect—both in the immediate and in the long term.

The OHIP-14 showed a statistically significant decrease in scores at all time points after whitening compared to baseline, although there is a slight regression of the values at 1 year, it still has a significant difference with the baseline. This decrease indicates that whitening produces a substantial improvement in the perception of patients and a noticeable reduction in all dimensions of physical, psychological, and social disability. These values significantly decreased with treatment providing important biopsychosocial implications. Usually, these perceptions of physical, mental, and social impairment are experienced by people with cosmetic dental problems and can profoundly affect their self-esteem, interactions, environmental adaptations, relationships, personal, job opportunities, and fundamental aspects that affect the quality of life [34].

To experience any positive change after the whitening treatment, patients require interaction with their social environment [35]. In the current study, the results were perceived soon after the treatment. Moreover, since all dimensions in the OHIP-14 at one-year post whitening kept better than baseline suggests that the psychosocial results were not only immediate but also had a long-term effect.

The limited available literature on the self-perception of the aesthetics and psychosocial impact generated by teeth whitening has shown psychosocial changes resulting from patients’ self-perception of aesthetics [12, 15, 18]. Nevertheless, more research is needed to support these results more conclusively. On the other hand, even though simple and short-term interventions do not affect personality factors [36], it can be emphasized that there is an impact on psychosocial factors and personal perception, intervening positively on the patient’s self-esteem and finally on their health status.

This study was limited by a loss to follow-up since the droput was greater than expected (only 29 patients at 1 year follow-up, 3 less than expected).. One limitation of measuring with instruments such as spectofotometer can be a discrete measurement of reflectance data, edge-loss, i.e., especially when it comes to measuring tooth-shaped specimens, and should be considered as bias in color assessment trials.

Finally, it is advisable that future research also studied the changes in the psychosocial well being of patients subjected to at home whitening with different concentrations of gel, and to assess the effects of tooth whitening better, the psychosocial impact of whitening could be compared to untreated patients.

## Conclusions

Low (6%) and traditional concentrations of hydrogen peroxide gels (37.5%) were effective and stable at one-year post bleaching, even though their effectiveness was statistically different. Both treatments kept a positive effect on psychosocial and self-perception during a year of follow-up.

## Data Availability

Data available from: https://drive.google.com/file/d/1eX6U4U0ru-gUPdwU8HOJ73pFgtgm_-7z/view?usp=sharing

## Abbreviations

WIO: Whiteness formula
WIC: CIE whiteness formula
CONSORT: Consolidated Standards of Reporting Trials
SGU: Scale Guide Unit
CIE: Commission Internationalede L’Eclairag
PIDAQ: Psychosocial impact dental aesthetic questionnaire
OHIP-14: Oral health impact profile
FDI: World Dental Federation
